# Fast sensorless collision detection for resource-constrained pmsm controllers using an FFRLS-based method

**DOI:** 10.1038/s41598-026-43846-5

**Published:** 2026-03-09

**Authors:** Duo Zhao, Thai Ren, Ganke Huang, Minyu Liu

**Affiliations:** 1 School of Integrated Circuits Science and Engineering, Southwest Jiaotong University, Chengdu, 610000 China; 2 School of Electrical Engineering, Southwest Jiaotong University, Chengdu, 610000 China

**Keywords:** Sensorless collision detection, PMSM, FFRLS, Load torque identifier, Engineering, Mathematics and computing

## Abstract

This paper proposes a low-complexity, fast sensorless collision detection method for permanent-magnet synchronous motors (PMSMs) based on a forgetting-factor recursive least-squares (FFRLS) estimator. The method estimates the load torque from the motor motion equations and detects collisions by monitoring abrupt changes in a processed load-torque metric. The algorithm is tailored for resource-constrained embedded controllers: it avoids high-order matrix operations and the use of external sensors while achieving rapid detection. Experimental results under constant speed, acceleration, and time-varying load conditions demonstrate fast and repeatable detection performance. The approach provides a practical trade-off between computational cost and detection reliability for embedded motor drives.

## Introduction

Permanent magnet synchronous motors (PMSMs) provide multiple pole pairs, enabling high torque density while maintaining a low overall mass. With the advances in permanent magnet materials, power electronics, and position sensing technologies, the performance of PMSMs has been continuously improved. Nonlinearity, coupling, and multivariable dynamics are typical features of PMSMs, and their control systems are prone to disturbances–including external disturbances and internal parameter variations. As PMSMs gain widespread application in robotics, electric vehicles, and industrial automation, research interest in sensorless collision detection for PMSMs has grown rapidly–particularly for collaborative robots^1^.

In recent years, collision detection research has increasingly emphasized sensorless approaches. Such methods aim to reduce the added cost and complexity associated with physical sensors while enhancing overall system robustness. For robotic arms, classical studies on collision detection traditionally begin with robot kinematics and dynamics: collisions are treated as fault behaviors of the drive system, and detection is achieved using actuator fault detection and isolation (FDI) techniques^2^. However, this approach may fail to detect transient collisions occurring over very short time intervals. In addition, some works employ the modified extended state observer (MESO) to provide a natural and efficient framework for collision detection and identification in robot systems–without requiring joint acceleration estimation or inversion of the inertia matrix. Compared with the well-established generalized momentum-based (GM) method, MESO yields more accurate collision force estimates under comparable noise levels in practice, albeit at the cost of a slightly higher computational load and increased observation delay^3^. Under a similar paradigm, high-order sliding mode observers^4^ and generalized momentum-based observers^5^ have also demonstrated strong performance in estimating joint motor load torque; however, their model construction processes remain relatively complex. Other studies achieve collision detection, impact point localization, and classification of collision nature—using either classical analytical methods or neural-network-based learning approaches^6^—though these typically demand substantial computing resources from the host computer.

A more concise alternative focuses directly on the joint drive motors of robots, inferring collision risk by analyzing motor current^7^. A collision is flagged upon detecting a sudden change in current; yet in practice, current-based methods suffer from poor accuracy due to measurement noise. Regardless of the specific technique employed, sensorless strategies share a common simplifying assumption: approximating the load torque as the motor’s electromagnetic torque. While this approximation streamlines implementation, it introduces modeling error. To address this limitation, certain studies adopt the model reference adaptive system (MRAS) to identify key parameters and construct a load torque observer using the Kalman filter—starting with estimation of load torque variations^8^. This improves fidelity over the electromagnetic-torque approximation, but the resulting algorithm involves multi-step iterative computations, rendering it unsuitable for low-cost, resource-constrained motor controllers.

In summary, although existing sensorless collision detection methods offer notable accuracy benefits, they face practical challenges in broader PMSM applications—such as conventional motion control scenarios in industrial automation, conveyor systems, and cost-sensitive controller deployments. In engineering practice, most robotic motor drives operate under strict cost constraints, relying on embedded controllers with limited word length and processing capability. Under such constraints, large-scale matrix operations, optimization algorithms, and AI-driven methods become infeasible. Hence, there is a clear need for a lightweight, fast response, low-complexity collision detection algorithm. The method proposed in this paper effectively addresses these requirements and is especially well-suited for embedded processors with limited computational resources.

The structure of this paper is as follows: Section “Methods” presents the theoretical foundation; Section “Experiments” details the experimental setup and validates the performance advantages of the proposed method; and Section “Conclusions” summarizes the work and outlines directions for future research.

## Modeling and collision detection strategy

### Mathematical model of the PMSM

For a real-time embedded controller that has a clear sample-time, we usually use a discrete model. The mechanical equation of the PMSM is:

1 $$\begin{aligned} J \cdot \frac{d\omega }{dt} = T_e - T_L - B \cdot \omega \end{aligned}$$

where *J* is the inertia. $\omega$ is the mechanical velocity. $T_e$ is the electromagnetic torque. $T_L$ is the load torque, which includes the possible collision torque. *B* is the viscosity coefficient. The complete mechanical model of a PMSM includes Coulomb friction and quadratic friction terms. However, for the purpose of estimator derivation and given the operating speed range considered in this study, a simplified linear viscous-friction model is adopted as shown in (1).

By performing a Laplace transform on (1), we have the following equation 2.

2 $$\begin{aligned} {T_e}\left( s \right) - {T_L}\left( s \right) = B \cdot \omega \left( s \right) + J \cdot s \cdot \omega \left( s \right) \end{aligned}$$

Let the above equation be expressed as: $U(s) = (B + J \cdot s) \cdot y(s)$, then the open-loop pulse transfer function of the mechanical motion equation (2) is as follows:

3 $$\begin{aligned} \frac{{H(s)}}{s} = \frac{1}{s} \cdot \frac{{y(s)}}{{u(s)}} = \frac{1}{s} \cdot \frac{{\frac{1}{J}}}{{s + \frac{B}{J}}} \end{aligned}$$

Performing the $\mathscr {Z}$ transform on the open-loop pulse transfer function (3) gives:

4 $$\begin{aligned} \mathscr {Z}\left( {\frac{{H(s)}}{s}} \right) = \frac{1}{B} \cdot \frac{1}{{z - 1}} \cdot \frac{{1 - {e^{ - \frac{B}{J}{T_s}}}}}{{1 - {z^{ - 1}} \cdot {e^{ - \frac{B}{J}{T_s}}}}} \end{aligned}$$

In addition, the transfer function of the zero-order holder is: ${G_0}(s) = \frac{{1 - {e^{ - {T_s} \cdot s}}}}{s}$. On the basis of the equivalence principle, the open-loop pulse transfer function with the zero-order holder can be derived as:

5 $$\begin{aligned} G(z) = \frac{{\omega (z)}}{{{T_e}(z) - {T_L}(z)}} = \frac{{{z^{ - 1}} \cdot (1 - {e^{ - \frac{B}{J}{T_s}}})}}{{B \cdot (1 - {z^{ - 1}} \cdot {e^{ - \frac{B}{J}{T_s}}})}} \end{aligned}$$

Taking the inverse $\mathscr {Z}$ transform of (5), the corresponding discrete-time difference equation can be written as

6 $$\begin{aligned} \omega (k) = \frac{1 - e^{-\frac{B}{J}T_s}}{B} \bigl (T_e(k-1) - T_L(k-1)\bigr ) + e^{-\frac{B}{J}T_s}\,\omega (k-1). \end{aligned}$$

In discrete-time systems, the angular velocity increment at time step *k* is defined as

7 $$\begin{aligned} \Delta \omega (k) = \omega (k) - \omega (k-1) \end{aligned}$$

For sufficiently small sampling periods, the exponential term in (6) can be approximated using a first-order Taylor expansion as

8 $$\begin{aligned} e^{-\frac{B}{J}T_s} \approx 1 - \frac{B}{J}T_s \end{aligned}$$

Substituting (8) into (6), rearranging terms, and neglecting higher-order terms yield,

9 $$\begin{aligned} \frac{\Delta \omega (k)}{T_s} = \frac{1}{J} T_e(k-1) - \frac{1}{J}\!\left( T_L(k-1) + B \omega (k-1)\right) \end{aligned}$$

which represents a first-order discrete-time approximation of the exact model in (6) and is equivalent to a forward Euler discretization of the continuous-time mechanical dynamics.

The above equation (9) is adopted for real-time implementation due to its simplicity. It can be rewritten in matrix form:

10 $$\begin{aligned} \frac{{\Delta \omega (k)}}{{{T_s}}} = {\left[ {\begin{array}{*{20}{c}}{{T_e}(k - 1)}\\ { - 1}\end{array}} \right] ^T} \cdot \left[ {\begin{array}{*{20}{c}}{\frac{1}{J}}\\ {\frac{1}{J} \cdot ({T_L}(k - 1) + B \cdot \omega (k - 1))}\end{array}} \right] \end{aligned}$$

For most surface-mounted PMSMs, since they lack salient pole characteristics, we can approximately assume that the rotor d-axis inductance is equal to the q-axis inductance in the stationary coordinate system, thus deriving the electromagnetic torque equation^9^:

11 $$\begin{aligned} T_e = \frac{3}{2}\cdot \frac{P}{2} \cdot {\psi _f} \cdot i_q \end{aligned}$$

Based on the motor’s known physical parameters–including the number of poles *P* and the armature flux linkage $\psi _f$–and by sampling and transforming the phase currents to obtain the q-axis current $i_q$ in the rotor-fixed rotating reference frame, the electromagnetic torque can be calculated using the torque equation (11).

### Parameter identification using FFRLS

This method employs the forgetting-factor recursive least-squares (FFRLS) algorithm to estimate the load torque. The principle of FFRLS is that the system continuously acquires new data and performs recursive parameter estimation; when the sum of squared errors is minimized, accurate estimation of the target parameters is achieved. FFRLS has been proven to guarantee exponential convergence of the parameter estimation error under the persistence-of-excitation condition and is applicable to multiple-input–multiple-output (MIMO) systems^10^.

As a mathematical identification method, FFRLS does not impose strict physical constraints; therefore, reducing the dimensionality of the estimated parameter vector generally enhances estimation accuracy. Moreover, it is essential to set physically meaningful bounds on the parameters to be estimated–consistent with the definition and physical range of the corresponding quantity.

Based on the FFRLS algorithm and Equation (10), define the input parameter sequence $\varphi (k)$, the parameter vector to be estimated $\hat{\theta }(k)$, and the output parameter sequence *y*(*k*) as follows:

12 $$\begin{aligned} \varphi (k) = \left[ {\begin{array}{*{20}{c}}{{T_e}(k - 1)}\\ { - 1}\end{array}} \right] \end{aligned}$$

13 $$\begin{aligned} \hat{\theta } (k) = \left[ {\begin{array}{*{20}{c}}a\\ b\end{array}} \right] \end{aligned}$$

14 $$\begin{aligned} y(k) = \frac{{\Delta \omega (k)}}{{{T_s}}} \end{aligned}$$

For (13) we have:

15 $$\begin{aligned} \left\{ {\begin{array}{l} a = \dfrac{1}{J}\\ b = a \cdot ({T_L}(k - 1) + B \cdot \omega (k - 1)) \end{array}} \right. \end{aligned}$$

It follows from the physical definition of the moment of inertia that parameter $a> 0$; furthermore, the physical quantity to be estimated can be calculated from the above equation (15):

16 $$\begin{aligned} \left\{ {\begin{array}{l} \hat{J} = \dfrac{1}{a}\\ {{\hat{T}}_L}(k - 1) = \dfrac{b}{a} - B \cdot \omega (k - 1) \end{array}} \right. \end{aligned}$$

The specific iterative update equations of the FFRLS algorithm are as follows:

17 $$\begin{aligned} \left\{ {\begin{array}{l} \hat{\theta } (k) = \hat{\theta } (k - 1) + K(k) \cdot [y(k) - {\varphi ^T}(k) \cdot \hat{\theta } (k - 1)]\\ P(k) = \dfrac{1}{\lambda } \cdot [I - K(k) \cdot {\varphi ^T}(k)] \cdot P(k - 1)\\ K(k) = \dfrac{{P(k - 1) \cdot \varphi (k)}}{{\lambda + {\varphi ^T}(k) \cdot P(k - 1) \cdot \varphi (k)}} \end{array}} \right. \end{aligned}$$

Here, *P*(*k*) denotes the covariance matrix, *K*(*k*) the gain vector (or gain matrix), and $\lambda$ the forgetting factor. The value of the forgetting factor must be tuned according to the specific implementation; it is typically chosen within the range $\lambda \in (0.9,\,1)$. In each sampling period, substituting Equations (12), (13), and (14) into the FFRLS update equation (17) yields the estimated parameter vector, from which the target physical quantity is then derived. Typically, the algorithm is initialized as follows:

18 $$\begin{aligned} \left\{ {\begin{array}{l} {P(0) = \beta \cdot I\;\;\;\;\beta \in (\begin{array}{*{20}{c}} {{{10}^2},}& {{{10}^6})} \end{array}}\\ {\hat{\theta } (0) = \varepsilon } \end{array}} \right. \end{aligned}$$

In Equation (18), $\beta$ is typically chosen as a sufficiently large positive real number, while $\epsilon$ is set to a sufficiently small positive real number or zero–yielding improved estimation performance.

**Fig. 1:**
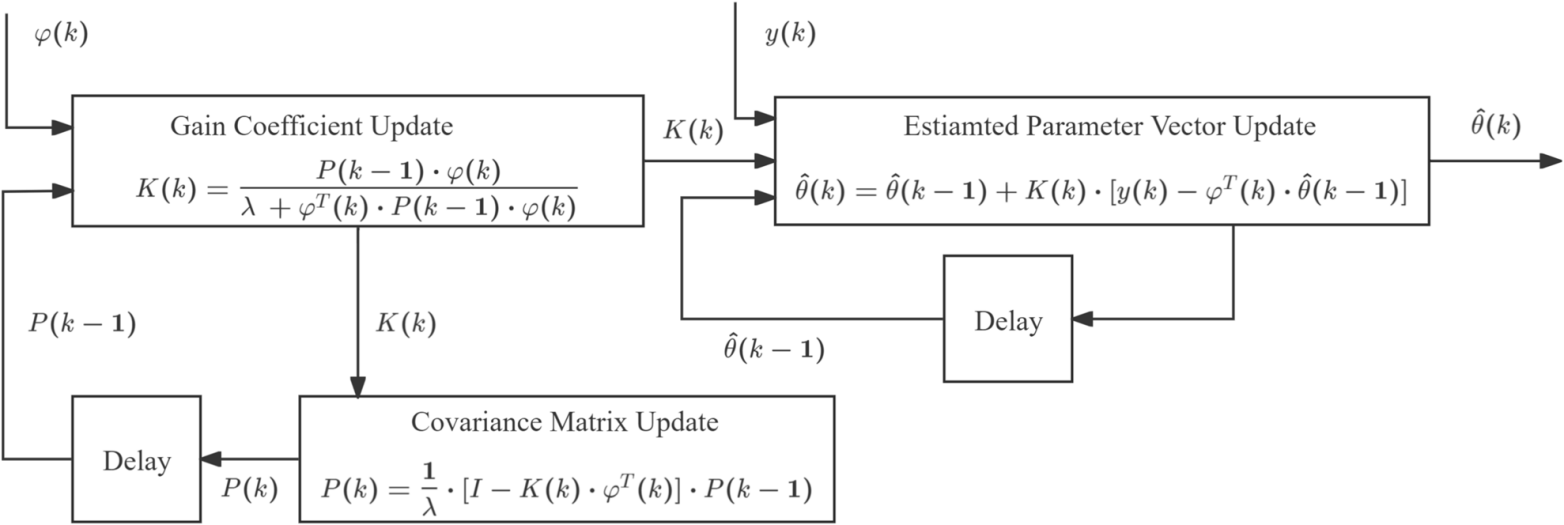
The parameter vector estimation process based on FFRLS.

### Collision detection

In the previous section, the load torque estimate $\hat{T}_L$ was obtained. In this work, a collision is detected when $\hat{T}_L$ exhibits a sudden change. Under ideal conditions, the forward difference could serve directly as the variation criterion–introducing an algorithmic delay of only two sampling periods. However, in practical embedded systems, various noise sources degrade the reliability of using this raw difference as the variation indicator. To suppress noise, we combine the central difference method with a moving-average filter to estimate abrupt load-torque changes. The discrete-time variation of the estimated load torque, denoted $\Delta \hat{T}_L(k)$, is defined as:

19 $$\begin{aligned} \Delta {\hat{T}_L}(k) = ({\hat{T}_L}(k) - {\hat{T}_L}(k - 2))/2 \end{aligned}$$

Because motors in applications such as robotics and robotic-arm joints frequently alternate between forward and reverse rotation, a speed-dependent correction term is introduced to prevent false positives. Let *D*(*k*) denote the evaluation value at discrete time step *k*. Since the raw metric has small magnitude, a numerical scaling factor $k_d$ is applied to enhance interpretability and simplify threshold tuning. The expression for *D*(*k*) is given by:

20 $$\begin{aligned} D(k) = k_d \cdot \Delta {\hat{T}_L}(k) \cdot \omega (k) \end{aligned}$$

The corresponding dynamic thresholds are defined as follows:

21 $$\begin{aligned} {\left\{ \begin{array}{ll} T_{\max } = T_0 + \left| \omega (k) \right| \cdot m \\ T_{\min } = -T_{\max } \end{array}\right. } \end{aligned}$$

where $T_{\max }$ and $T_{\min }$ denote the upper and lower threshold values, respectively. $T_0$ is the base threshold–set to a small positive value that can be tuned according to the motor’s rated torque. The coefficient *m* quantifies the speed-dependent scaling of the threshold: higher rotational speeds necessitate proportionally larger thresholds. This parameter is application-specific; it is typically set to a relatively small value in high-speed, low-sensitivity applications (e.g., conveyor belts, fans), whereas in low-speed, high-precision scenarios (e.g., robotic arms, robotic-joint drives), a larger *m* is used to enhance robustness against false positives.

To summarize, the judgment formula for collision detection is as follows:

22 $$\begin{aligned} \begin{array}{l} if\; D(k) \notin (\begin{array}{*{20}{c}} {{T_{\min }},}& {{T_{\max }}} \end{array}),\;then\;set\;Flag = 1\\ else\;set\;Flag = 0 \end{array} \end{aligned}$$

A transition of the *Flag* bit from 0 to 1 indicates that a collision has occurred.

## Experiments

### Introduction to the experimental platform and dataset

**Fig. 2:**
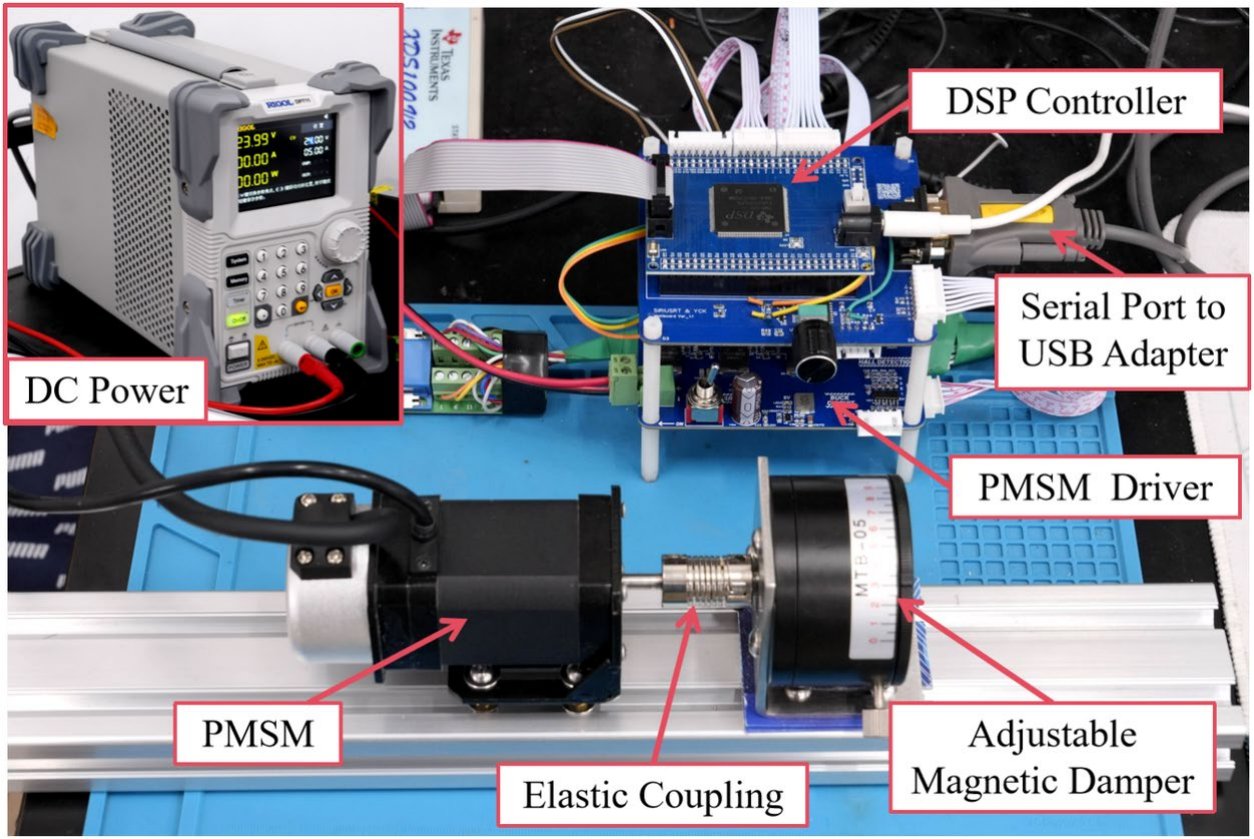
Experimental platform.

The experimental platform comprises the following components: (1) a PMSM (model: 42JSF630AS-1000, equipped with a 1000-line absolute encoder); (2) a DC power supply (model: RIGOL DP711); (3) a PMSM drive system based on a DSP controller (Texas Instruments TMS320F28335PGFA); (4) an elastic coupling; (5) an adjustable magnetic damper; and (6) a serial-to-USB adapter. The adjustable magnetic damper is a permanent-magnet brake that delivers an approximately constant braking torque across a wide speed range. Owing to its speed-independent torque characteristic, the damper is used to impose a stable and repeatable external load torque on the motor shaft. By adjusting the damper setting, various baseline load conditions can be established without modifying the control structure or operating mode of the drive system. See Table 1 for the PMSM’s detailed specifications. The experimental setup is illustrated in Fig. 2.

**Table 1:** Motor specification.

Specifications	Value	Specifications	Value
Phase	3	Motor Pole Pairs	4
Rated DC Voltage	24 V	PM Flux Linkage	7.797 $\times 10^{-3}$ Wb
Rated Torque	0.2 N $\cdot$ m	Viscous Damping	4.37 $\times 10^{-4}$ N $\cdot$ ms/rad
Rated Power	64 W	Rotor Inertia	2.8 $\times 10^{-6}$ kg $\cdot$ m^2^
Rated Current	4.0 A	Phase Resistance	0.54 ${\Omega }$ $\pm 10\%$
Rated Speed	3000 r/min	Phase Inductance	0.62 $\times 10^{-3}$ H $\pm 20\%$

The sampling frequency of the embedded controller is set to 12500 Hz, and it communicates with the host computer via the SCI (Serial Communication Interface) port. Due to hardware limitations of the host computer’s experimental equipment, the real-time serial communication rate is limited to 2500 Hz to prevent loss of synchronization. The motor experimental platform employs a PI-based closed-loop control structure, with the speed loop operating at 2500 Hz. In conjunction with space vector pulse width modulation (SVPWM), the PMSM is driven. All experiments were conducted using a unified cascaded speed–current control architecture. No external torque or current reference was injected during the tests; the *q*-axis current reference was generated solely by the speed controller based on the speed tracking error.

Through repeated experiments, the algorithm parameters are determined as follows: the forgetting factor $\lambda$ is set to 0.95; $\beta = 100$; and $\epsilon = 0$. Based on the magnitude of $\Delta \hat{T}_L(k) \cdot \omega (k)$ in (20), a scaling factor $k_d = 10^4$ is adopted to facilitate observation and threshold tuning. Additionally, the window length of the moving average filter applied to the estimated load torque $\hat{T}_L$ is set to $L = 10$, which provides a favorable trade-off between noise suppression and time delay.

The initial value of the speed influence factor *m* is determined via a speed step-response experiment. After system power-up and initialization, the controller operates until the FFRLS algorithm achieves steady-state convergence. Subsequently, a step command is applied to the speed reference, starting from an initial operating point corresponding to the rated speed. During this transient process, the time interval from the onset of rotor acceleration $t_0$ to the moment $t_s$ when the rotational speed reaches the rated value $\omega _{\text {rated}}$ (within a tolerance of $\pm 2\%$) is recorded. Concurrently, the evaluation value *D*(*k*) is monitored, and its maximum value $D_{\text {max}}$ within this interval–along with the corresponding time instant $t_{\text {eva}}$–is extracted. A margin coefficient $K_m$ is introduced to adjust the sensitivity of collision detection. In this work, $K_m = 2$ is used, although a practical range of $K_m \in [1.5,\,3.0]$ is recommended. Based on these measurements, the initial value $m_0$ of the speed influence factor is computed using Equation (23).

23 $$\begin{aligned} { m_0 = \frac{E_{\text {max}} \cdot (t_s - t_0)}{\omega _{\text {rated}} \cdot (t_{\text {eva}} - t_0)} \cdot K_m } \end{aligned}$$

The base threshold $T_0$ is obtained during steady-state operation at rated speed. After the motor has operated at rated speed for a sufficiently long duration, the evaluation value *D*(*k*) is monitored over a short time window (typically $5-10s$). The maximum peak value observed within this interval can be selected as $T_0$, representing the basic value of normal operating variations in the absence of external disturbances. The base threshold $T_0$ also serves as a robustness margin to suppress false detections caused by measurement noise and normal dynamic load variations. In this study, the speed influence factor after amplification is set to $m = 0.15$, and the minimum basic threshold $T_0 = 10$.

Note that, because FFRLS is an estimation algorithm, residual estimation errors may persist even after convergence. However, motor parameters have well-defined physical meanings; therefore, it is essential to impose physically meaningful bounds on the estimated parameters at each iteration in practical implementations. The covariance update *P*(*k*) involves computationally expensive matrix operations and is not required in all deployment scenarios. In practice, *P*(*k*) is primarily used as a convergence diagnostic during algorithm debugging. In a fully debugged system, the computation of *P*(*k*) can be omitted, which enhances the practicality and usability of the proposed method.

### Collision test

To verify the effectiveness of the proposed collision detection algorithm, the following experiments were conducted on the experimental platform: (1) constant-speed loaded collision test; (2) accelerated-speed loaded collision test; and (3) variable-load collision test.

In all collision tests, the term “initial load torque” refers to the steady-state external torque applied by the magnetic damper prior to collision onset. This torque constitutes a constant mechanical disturbance acting on the motor shaft–compensated by the cascaded speed–current control loop during normal operation–and serves as the baseline operating condition for collision detection.

In the experiments, friction pads were used to simulate sudden load changes during collision. Collision events were emulated by applying a short-duration additional torque to the shaft coupling via a friction pad. This method introduces an abrupt and irregular external disturbance that approximates the stochastic nature of real physical collisions. The magnitude of the applied collision torque was estimated–based on repeated experimental observations–to range from approximately 0.004 N $\cdot$ m to 0.015 N $\cdot$ m. The torque pulse duration was less than 0.1 s, after which it was fully removed. During the collision tests, both the initial load torque and the applied collision torque were purely external mechanical disturbances–not control inputs–and were compensated by the speed controller through corresponding adjustments of the *q*-axis current reference.

**Fig. 3:**
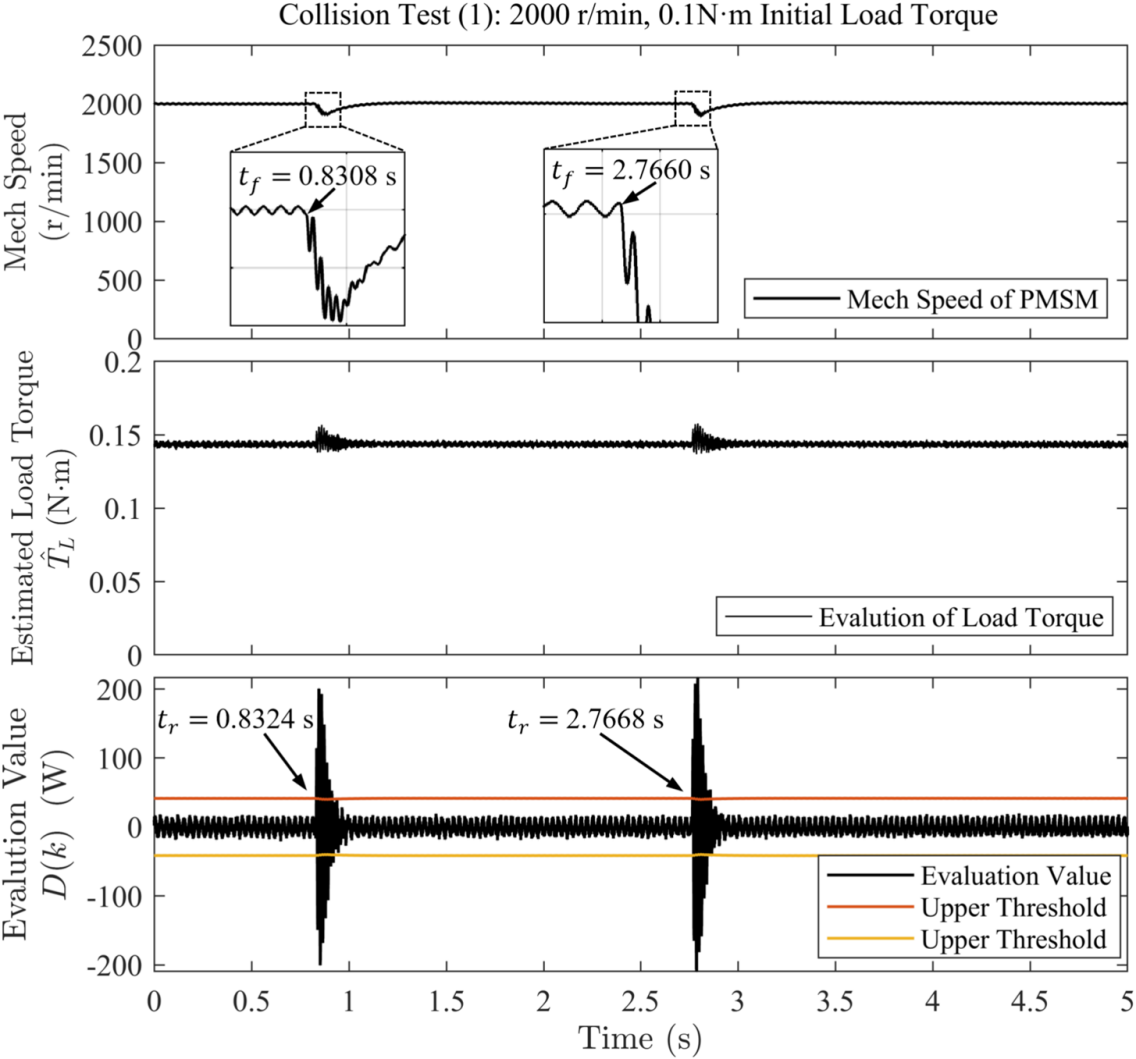
Waveforms of rotational speed and evaluation value under sudden load application in collision test (1).

**Fig. 4:**
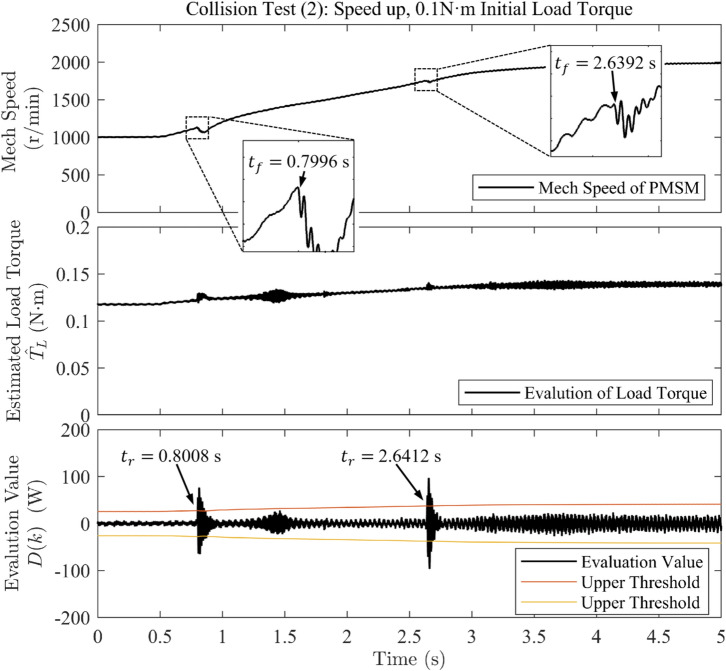
Waveforms of rotational speed and evaluation value under sudden load application when accelerating in collision test (2).

**Fig. 5:**
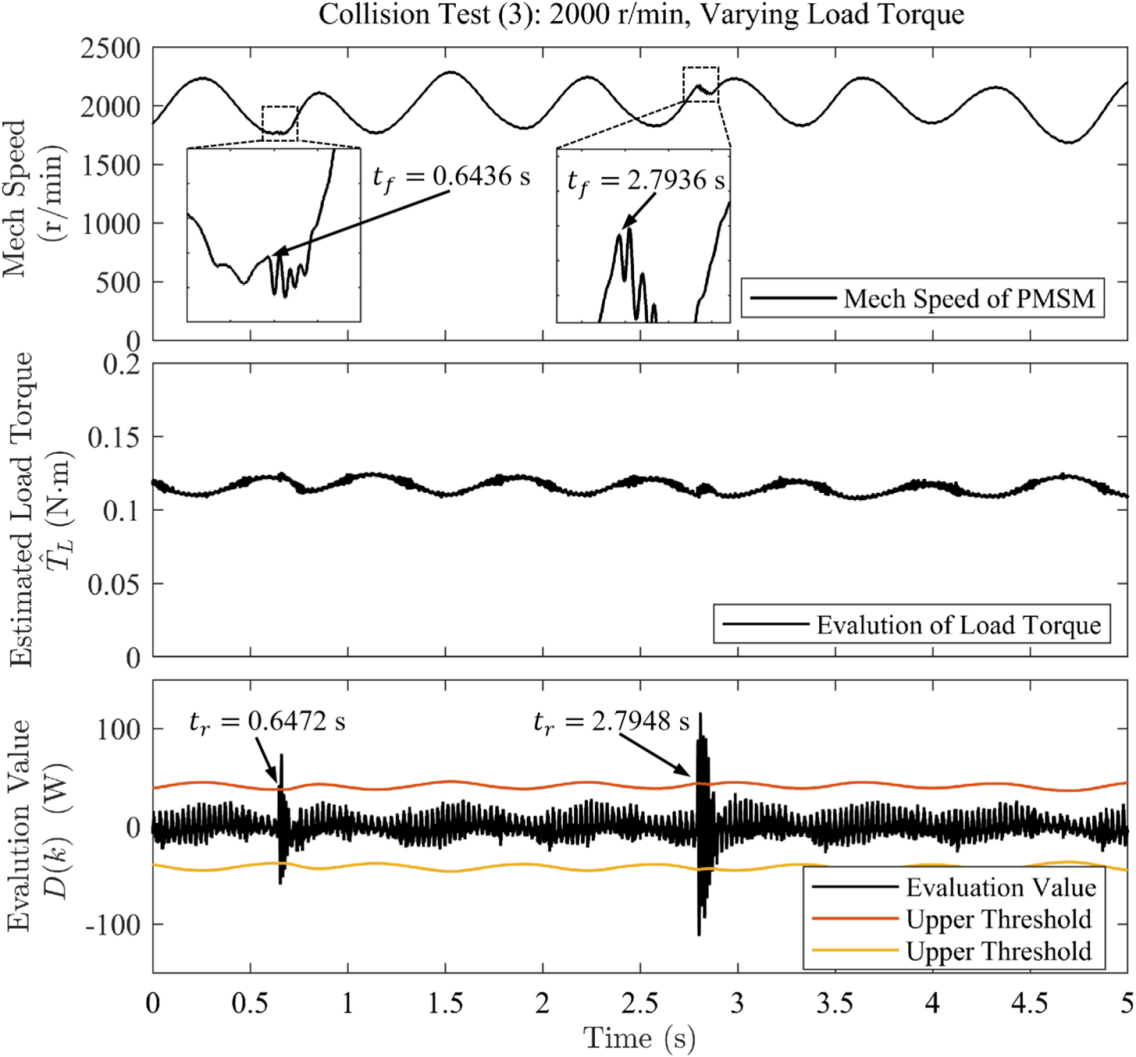
Waveforms of rotational speed and evaluation value under sudden load application when accelerated in collision test (3).

For each experiment, two collision events were induced to improve result reliability, with an interval of at least 1 second between them. For test (1), the motor’s mechanical speed reference is held constant at 2000 r/min, and the initial load torque is set to 0.1 N $\cdot$ m. For test (2), the speed reference starts at 1000 r/min and ramps linearly to 2000 r/min, while the initial load torque remains constant at 0.1 N $\cdot$ m. For test (3), the speed reference is held constant at 2000 r/min, and the load torque is varied sinusoidally between 0.05 N $\cdot$ m and 0.1 N $\cdot$ m at a frequency of approximately 1.5 Hz. In Figures 3, 4, and 5, motor mechanical speed is reported in r/min for clarity; all analytical derivations in this work use angular speed $\omega (k)$ expressed in rad/s.

The detection time of the algorithm is defined as the interval from the onset of speed decrease ($t_f$) to the first instant when the evaluation value exceeds the threshold ($t_r$), as observed in the speed and evaluation-value waveforms on the host computer.

The collision test results obtained from experimental data analysis are summarized in Table 2. The results show that, in Test (1), the controller detected the collision within 1.6 ms and 0.8 ms, respectively–fast detection times that provide additional time redundancy for post-collision protection measures. In Test (2), the strategy also exhibited rapid response during motor acceleration, with detection times of 1.2 ms and 2 ms for the two collisions. In Test (3), even under continuously varying load torque, the strategy achieved a detection time of 3.6 ms for the first, milder collision, whereas the detection time was only 0.8 ms for the second, more severe collision.

**Table 2:** Collision test results.

Collision test	Additional collision Torque (N $\cdot$ m)	Detection time (ms)
Test (1)	0.0135	1.6
	0.0144	0.8
Test (2)	0.0081	1.2
	0.0064	2.0
Test (3)	0.0046	3.6
	0.0083	0.8

To evaluate the robustness of the proposed collision detection strategy against temperature-induced parameter variations, additional experiments were conducted under different thermal conditions using the constant-speed loaded collision test (Test (1)). This test was selected as a representative scenario because it effectively isolates thermal effects from acceleration-related dynamics.

Collision Tests (4) and (5) were performed under near-room-temperature and elevated-temperature conditions, respectively. In the near-room-temperature case, the measured motor surface temperature was approximately 34.8 $^{\circ }$C. For the elevated-temperature case, the experiment was conducted after the motor reached a steady thermal state with a maximum surface temperature of approximately 80.8 $^{\circ }$C, as measured using an infrared thermal imaging camera. The corresponding thermal images are available in the Supplementary Materials. In both experiments, the motor was operated at a constant speed of 2000 r/min with an initial load torque of 0.1 N $\cdot$ m.

All control parameters and experimental conditions were kept identical. The detection thresholds used in these experiments were identified based on the upgraded motor drive hardware platform and were kept unchanged across both temperature conditions, with $m_0 = 0.2$ and $T_0 = 15$.

**Fig. 6:**
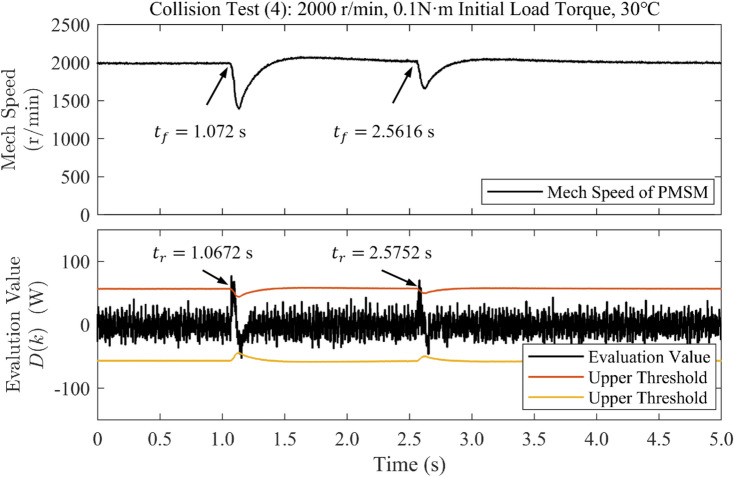
Waveforms of rotational speed and evaluation value under sudden load application when accelerated in collision test (4) at approximately 30 $^\circ$C.

**Fig. 7:**
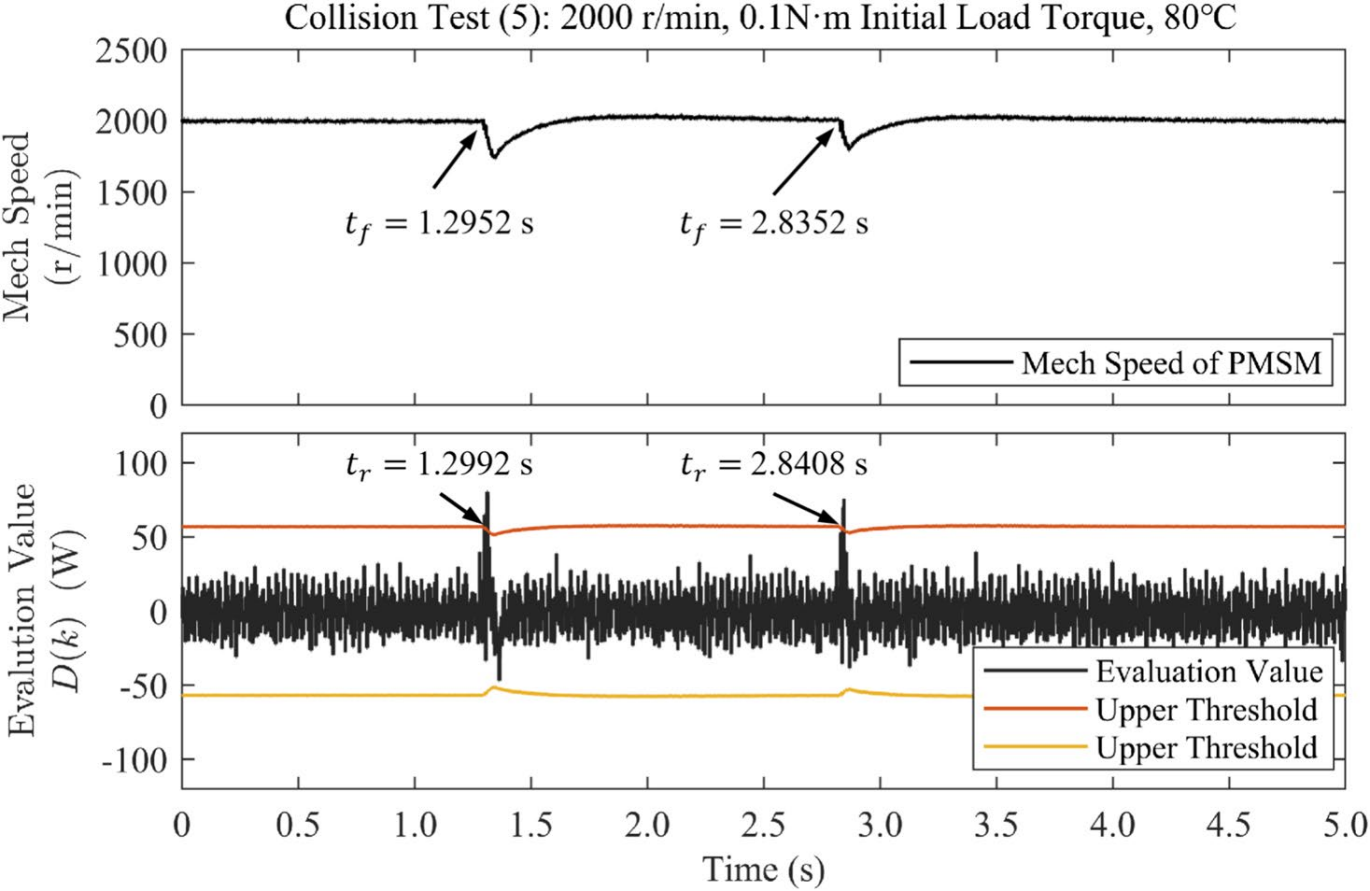
Waveforms of rotational speed and evaluation value under sudden load application when accelerated in collision test (5) at approximately 80 $^\circ$C.

Figures 6 and 7 show the measured rotational speed and evaluation value under sudden load application at approximately 30 $^{\circ }$C and 80 $^{\circ }$C, respectively. As shown in Fig. 6, two collision events were detected within 5.6 ms and 4.8 ms under near-room-temperature conditions. In the elevated-temperature case shown in Fig. 7, the corresponding detection times were 4.0 ms and 5.6 ms.

These results demonstrate that the proposed collision detection method maintains reliable and timely detection performance over a wide temperature range. The method is therefore robust against moderate temperature-induced variations in motor parameters, such as permanent-magnet flux linkage and viscous damping, which are commonly encountered in practical industrial applications.

The experiments were designed to evaluate the algorithm’s ability to detect the onset of abrupt external disturbances; precise measurement of collision torque was outside the scope of this study. The proposed detector identified collisions induced by additional torques as small as 0.0046 N $\cdot$ m, which is substantially lower than the initial load torque used in the tests. Moreover, repeated experiments and data analysis show that larger applied collision torques correlate with shorter algorithmic detection times. The detector exhibits robust detection speed across diverse motor operating conditions and provides a rapid, reliable trigger signal for post-collision control strategies.

## Conclusion

This paper proposes a sensorless collision detection strategy for PMSMs based on the FFRLS algorithm. The proposed method requires neither additional sensors nor a state observer model. By performing iterative updates on low-dimensional matrices derived from discretized motor motion equations, it achieves accurate and responsive tracking of load torque variations. Consequently, the method is well suited for embedded processors with limited word length and computational resources. In this study, collision tests were conducted under three operating conditions: constant-speed, variable-speed, and variable-load-torque scenarios. These experiments validate the high performance and robustness of the proposed approach. FFRLS serves as the core load torque estimator; its theoretical minimum detection time in simulation is only two sampling periods. Due to hardware limitations of the experimental platform, digital filters were employed to suppress signal noise–introducing a small but non-negligible delay. Therefore, we expect superior detection performance on a well-engineered drive system platform.

## Discussion

The experimental setup does not include an independent torque sensor for direct measurement of the external load torque. Therefore, the absolute accuracy of the estimated torque $\hat{T}_L$ cannot be quantitatively validated. However, the objective of the proposed strategy is reliable collision detection rather than precise torque identification. The detection mechanism depends on transient variations of the estimated torque and its evaluation metric, rather than on the exact steady-state value of $\hat{T}_L$. Future work may incorporate high-precision torque sensing to further evaluate estimation accuracy.

Because convergence of parameter estimation errors in the FFRLS algorithm requires persistent excitation and sufficient time, accumulated excitation is insufficient during system initialization–leading to a non-negligible risk of false detection at the initial stage. To prevent unreliable decisions during this phase, collision judgment is temporarily inhibited, introducing a short initialization “blind spot”. However, the algorithm achieves rapid convergence shortly after system startup. Experimental results indicate that convergence is achieved within approximately 120 ms on the experimental platform.

In practical applications, additional disturbances–including current-sampling noise, inverter switching harmonics, encoder quantization effects, and temperature-dependent parameter variations–may affect estimation accuracy. Although the proposed method demonstrates robustness under the tested conditions, further enhancement could be achieved by integrating adaptive or hybrid estimation frameworks, such as combining FFRLS with an extended Kalman filter, to improve disturbance rejection capability.

## Supplementary Information

Supplementary Information 1.

Supplementary Information 2.

## Data Availability

All data generated or analysed during this study are included in this published article and its supplementary information files. A simulation model for strategy verification is also included in the supplementary information files.
