# Supplementary material for: Fast sensorless collision detection for resource-constrained pmsm controllers using an FFRLS-based method
Source: Sci Rep. 2026 Mar 9;16:12667. doi: 10.1038/s41598-026-43846-5 (PMC13087039; doi:10.1038/s41598-026-43846-5)
Supplement: Supplementary file 2 — Supplementary Information 2. [file 41598_2026_43846_MOESM2_ESM.zip › Pseudocode_Box_for_SR_Reviewers.pdf]

Algorithm 1 presents the pseudocode box of FFRLS and collision detector.

Algorithm 1: FFRLS with Covariance Update

Input : Measurement $y(k)$, regressor $\varphi(k) \in \mathbb{R}^n$, forgetting factor λ

Output: Estimated parameters $\hat{\theta}(k)$, Collision flag

Initialization($k = 0$):

$\hat{\theta}(0) = \varepsilon, \quad P(0) = \beta \cdot I \quad \beta \in (10^2, 10^6) \ , \quad \lambda \in (0.9, 1)$

$\varphi(k) = \begin{bmatrix} T_e(k-1) \\ \\ -1 \end{bmatrix} \quad y(k) = \frac{\Delta\omega(k)}{T_s}$

for $k = 1, 2, \ldots$ **do**

(1) Innovation computation

$\varepsilon(k) \leftarrow y(k) - \varphi(k)^\top \hat{\theta}(k-1)$

(2) Gain vector update (no matrix inverse)

$$K(k) \leftarrow \frac{P(k-1) \cdot \varphi(k)}{\lambda + \varphi^T(k) \cdot P(k-1) \cdot \varphi(k)}$$

(3) Parameter update

$\hat{\theta}(k) \leftarrow \hat{\theta}(k-1) + K(k)\,\varepsilon(k)$

(4) Covariance update

$$P(k) \leftarrow \text{Symmetric Regularization} \leftarrow \frac{1}{\lambda} \cdot [I - K(k) \cdot \varphi^T(k)] \cdot P(k-1)$$

(5) Collision detection (scalar operations only)

Extract $\widehat{T}_L(k)$ from $\hat{\theta}(k)$, Moving Average Filter($\widehat{T}_L(k)$)

$\Delta\hat{T}_L(k) \leftarrow (\hat{T}_L(k) - \hat{T}_L(k-2))/2$

$D(k) \leftarrow \Delta\hat{T}_L(k) \cdot \omega(k)$

if $D(k) \notin [T_{\max}, T_{\min}]$ **then**

Collision_Flag $\leftarrow$ true

else

Collision_Flag $\leftarrow$ false

Computational Properties:

- Covariance update processes a rank-one outer product and a second order matrix multiplication, *no matrix inversion required.* To ensure the convergence of FFRLS, the symmetrical regularization is necessary.
- Per-step complexity is $O(n^2)$, with $n = 2$ in this work.
- Only scalar filtering and comparisons are used for collision decision.
