# Supplementary figures and images for: Fast sensorless collision detection for resource-constrained pmsm controllers using an FFRLS-based method
Source: Sci Rep. 2026 Mar 9;16:12667. doi: 10.1038/s41598-026-43846-5 (PMC13087039; doi:10.1038/s41598-026-43846-5)

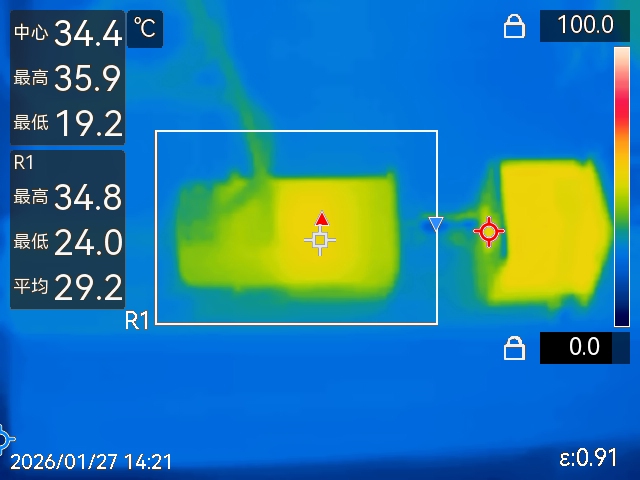

Supplement: Supplementary file 2 — Supplementary Information 2. [file 41598_2026_43846_MOESM2_ESM.zip › Thermal images/CollisionTest4_Thermal imaging.jpeg]

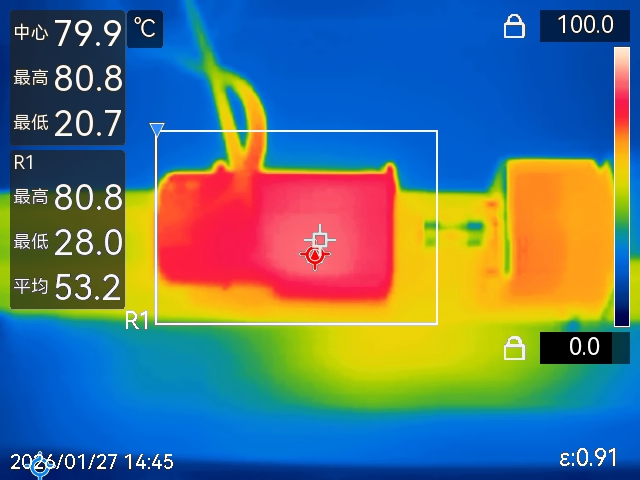

Supplement: Supplementary file 2 — Supplementary Information 2. [file 41598_2026_43846_MOESM2_ESM.zip › Thermal images/CollisionTest5_Thermal imaging.jpeg]
